# High-resolution magic angle spinning magnetic resonance spectroscopy detects glycine as a biomarker in brain tumors

**DOI:** 10.3892/ijo_00000500

**Published:** 2010-02-01

**Authors:** VALERIA RIGHI, OVIDIU C. ANDRONESI, DIONYSSIOS MINTZOPOULOS, PETER M. BLACK, A. ARIA TZIKA

**Affiliations:** 1 NMR Surgical Laboratory, Department of Surgery, Harvard Medical School and Massachusetts General Hospital, Boston, MA 02114;; 2 Athinoula A. Martinos Center of Biomedical Imaging, Department of Radiology, Massachusetts General Hospital, Boston, MA 02114;; 3 Department of Neurosurgery Brigham and Women’s Hospital, Harvard Medical School, Boston, MA 02115, USA

**Keywords:** brain/CNS cancers, tumor biomarkers, *ex vivo* high-resolution magic angle spinning magnetic resonance spectroscopy

## Abstract

The non-essential amino acid neurotransmitter glycine (Gly) may serve as a biomarker for brain tumors. Using 36 biopsies from patients with brain tumors [12 glioblastoma multiforme (GBM); 10 low-grade (LG), including 7 schwannoma and 3 pylocytic astrocytoma; 7 meningioma (MN); 7 brain metastases (MT), including 3 adenocarcinoma and 4 breast cancer] and 9 control biopsies from patients undergoing surgery for epilepsy, we tested the hypothesis that the presence of glycine may distinguish among these brain tumor types. Using high-resolution magic angle spinning (HRMAS) ^1^H magnetic resonance spectroscopy (MRS), we determined a theoretically optimum echo time (TE) of 50 ms for distinguishing Gly signals from overlapping myo-inositol (Myo) signals and tested our methodology in phantom and biopsy specimens. Quantitative analysis revealed higher levels of Gly in tumor biopsies (all combined) relative to controls; Gly levels were significantly elevated in LG, MT and GBM biopsies (P≤0.05). Residual Myo levels were elevated in LG and MT and reduced in MN and GBM (P<0.05 vs. control levels). We observed higher levels of Gly in GBM as compared to LG tumors (P=0.05). Meanwhile, although Gly levels in GBM and MT did not differ significantly from each other, the Gly:Myo ratio did distinguish GBM from MT (P<0.003) and from all other groups, a distinction that has not been adequately made previously. We conclude from these findings that Gly can serve as a biomarker for brain tumors and that the Gly:Myo ratio may be a useful index for brain tumor classification.

## Introduction

Thus far, brain tumor studies using proton (^1^H) magnetic resonance spectroscopy (MRS) have focused mainly on the absolute concentrations of metabolites, such as choline containing compounds (Cho), *N*-acetyl aspartate (NAA), and creatine (Cr), and on their concentration ratios (e.g., Cho/NAA or Cho/Cr) (1–3). However, there are other metabolites present in varying concentrations in brain tumors that may provide useful diagnostic information (4). Indeed a recent *in vivo* ^1^H MRS study reported significantly increased levels of glycine (Gly) in gliomas (5).

The non-essential amino acid Gly is an important neuro-transmitter and neuromodulator in the mammalian brain (6). It also has neurotrophic effects (7) and modulates the metabolism of microglial cells (8). High levels of Gly have been detected in brain tumors, especially in glioblastoma multiforme (GBM) tumors, as well as in the brains of patients with hyper-glycinemia (3,9–11). In addition, several studies have evaluated Gly as a potential treatment for schizophrenia (12–14). While the precise role of Gly in brain tumors is not clear, its presence is fitting with the hyper-excitable nature of brain tumors. Given Gly’s significance, it would be valuable to have an accurate measure of Gly in the brain. However such measurements are difficult because of the presence of Myo-inositol (Myo), a compound involved in signaling and in synthesis of inositol-containing-phospholipids (15,16), since Myo resonances overlap with the CH_2_-Gly singlet.

Selective Myo and Gly measurement methodologies using different *in vivo* ^1^H MRS filtering techniques have been described. For example, two-dimensional (2D) J-point resolved spectroscopy (PRESS) (17) and multiple refocusing pulses with a very long echo time (TE) (3) have been proposed for 3 T studies. A TE-averaged PRESS sequence (18) has also been proposed for 4 T studies. Other methods based on the differential signal dephasing of Gly and Myo metabolites have been proposed (19). Mader *et al*(20) observed a signal at 3.56 ppm attributable to Gly in long TE spectra and suggested that discrimination between tumor tissue from patients with astrocytoma grade II and GBM could be based on the greater levels of Gly in GBM. Although Mader *et al* results were corroborated (21), other authors using *in vivo* ^1^H MRS examinations of gliomas did not attempt to distinguish between Gly and Myo and interpreted their data as representing a rise in Myo (1,22). Indeed increased Myo concentrations were recently reported for a variety of tumors (23).

Previous studies have indicated that that a reliable separation between GBM and metastases cannot be confidently achieved with ^1^H MRS alone (24–26). Here, we used *ex vivo* high-resolution magic angle spinning (HRMAS) ^1^H MRS in order to evaluate the theoretically optimum echo time (TE) to enable Gly to be distinguished from overlapping Myo signals and tested our methodology in phantom and biopsy specimens. We focused on the Gly and Myo signals and acquired MR spectra with different TE spectra in order to discriminate amongst different tumor types.

## Materials and methods

### Simulated spectra

The simulated spectra were obtained using the XWINNMR software environment (XWINNMR version 3.5, Bruker Biospin) under Carr-Purcell-Meiboom-Gill (CPMG) (27) excitation.

### Phantom

*In vitro* HRMAS experiments were performed on a phantom sample (pH 7.0) containing 200 mM Myo and 200 mM Gly in deuterated water (Sigma Aldrich Inc., St. Louis, MO).

### Samples

Forty-five control biopsy samples from 9 epileptic surgeries and 36 tumor biopsies were analyzed. The tumor biopsies were derived from 12 GBM cases, 10 low-grade (LG) cases (7 schwannoma and 3 pylocytic astrocytoma), 7 meningioma (MN) cases, and 7 brain metastases (MT) cases (3 from adenocarcinoma and 4 from breast cancer). Subjects ranged in age from 17 to 54 years. This study was approved by our institutional review board.

### HRMAS ^1^H MRS acquisition data

Experiments were performed on a Bruker Bio-Spin Avance NMR spectrometer (600.13 MHz) using a 4-mm triple resonance (^1^H, ^13^C, ^2^H) HRMAS probe (Bruker). Specimens were pre-weighed and transferred to a ZrO_2_ rotor tube (4 mm diameter, 50 *μ*l), 10 *μ*l of external standard [trimethylsilyl propionic-2,2,3,3-d4 acid (TSP), Mw = 172, δ = 0.00 ppm) was added and functioned as a reference both for both resonance chemical shift and quantification. The HRMAS ^1^H MRS was performed at 3 kHz MAS speed and −8°C (the minimum temperature possible to minimize tissue degradation). One dimensional (1-D) water suppressed, fully relaxed spectra were acquired with an optimized rotor synchronized Carr-Purcell-Meiboom-Gill (CPMG) pulse sequence [90-(τ-180-τ)_n_-acquisition] (27). CPMG is preferred over simple free induction decays (FIDs) since it acts as a T2 filter that reduces the interference of very broad features in the spectrum baseline, originating from tissue water and macromolecules. CPMG sequence parameters were as follows: inter-pulse delay τ = 2π/ω_r_ = 400 *μ*s; 256 transients; spectral width of 7.2 kHz; 8 k data-points; and TR = 3 s. For quantification, we measured the T2 relaxation time by varying the CPMG evolution time (T_CPMG_ = 2nτ) [n from 7 to 800 (∼5–480 ms)].

### ^1^H HRMAS MRS data processing

MR spectra of specimens were analyzed using MestReC software (Mestrelab Research, www.mestrelab.com). A line-broadening apodization function of 1.0 Hz was applied to CPMG HRMAS ^1^H FIDs prior to Fourier transformation (FT). MR spectra were referenced with respect to TSP at δ = 0.0 ppm (external standard), manually phased, and a Whittaker baseline estimator was applied to subtract the broad components of the baseline.

### MRS-derived metabolite quantification

Concentrations of Gly and Myo metabolites were calculated using MestReC software (Mestrelab Research, http://www.mestrelab.com). An automated fitting routine based on the Levenberg-Marquardt algorithm (28,29) was applied after manual peak selection, adjusting peak positions, intensities, linewidths and Lorentzian/Gaussian ratio until the residual spectrum was minimized. Metabolite concentration (in *μ*mol/g) was calculated using the following equation:

$$\frac{\text{Mass}\;\text{TSP}}{\text{Mol}\;\text{weight}\;(\mathrm{TSP})}\times \frac{\text{Met}\;\text{peak}\;\text{area}}{\text{TSP}\;\text{peak}\;\text{area}}\times \frac{N_{\mathrm{TSP}}}{N_{\mathrm{Met}}}\times \frac{1000\text{g}/\text{kg}}{\text{Sample}\;\text{weight}}$$

where mass TSP was constant (*μ*g), the molecular weight (mol weight) of TSP was 172.23 g/mol; Met, metabolites; N_TSP_ was the TSP proton number (9 ^1^H), and N_Met_ was the metabolite proton number (30).

### Statistical analysis

Statistical analysis was performed using the Student’s t-test: paired two sample for means. P-values <0.05 were considered statistically significant.

## Results

### Theoretical

**Figure 1:**
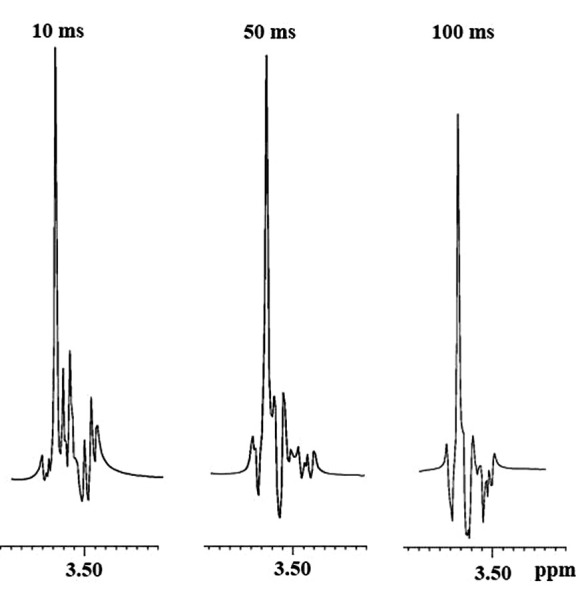
Simulated spectra of Gly (3.55 ppm) and Myo (3.53 ppm M2 H) under single spin-echo excitation. From left to right: simulated spectral line shape at TEs of 10, 50 and 100 ms. Spectra were simulated in the XWINNMR software environment.

Six CH groups form the Myo metabolite generate a complex spectral pattern in the ^1^H spectrum and can be modeled as an AM2N2P spin system. According to the spin system, the six protons have different resonances: the M protons resonate at 3.53 ppm (M2), the N resonates at 3.61 ppm (N2), and the A and P protons resonate at 4.07 and 3.28 ppm, respectively. On the contrary, the two protons of Gly constitute a singlet at 3.55 ppm. The Gly singlet overlaps with the strongly coupled resonances of Myo at 3.53 ppm (M2). The J-coupling constant between the M2 and N2 protons is 9.9 Hz. As TE increases, J-coupling introduces a dephasing in the M2 resonance due to the large J-coupling constants, which can be exploited for detecting the Gly singlet (19). Fig. 1 shows ^1^H CPMG HRMAS simulated spectra acquired at varied TEs (single coherence). The spectrum acquired at TE = 10 ms did not exhibit J-modulation, but at TE = 50 ms, we detected a strong Gly peak and a reduction of the Myo doublet at 3.53 ppm without loss of signal. This was possible because of the large J-coupling (9.9 Hz) of the Myo proton signal at 3.53 ppm and its fast J-modulation. The two protons of Gly constitute a singlet at 3.55 ppm and, like all singlets, it does not have phase modulation during the spin-echo single excitation. When we used a single spin-echo coherence, the signal of Myo at 3.53 ppm underwent rapid J-modulation, and at TE = 100 ms, the intensity of the Myo resonance was strongly reduced. At longer TEs (i.e., TE = 300 ms), we detected the Gly singlet but observed a general reduction of the signal in the spectrum due to T2 relaxation.

### Experimental

**Figure 2:**
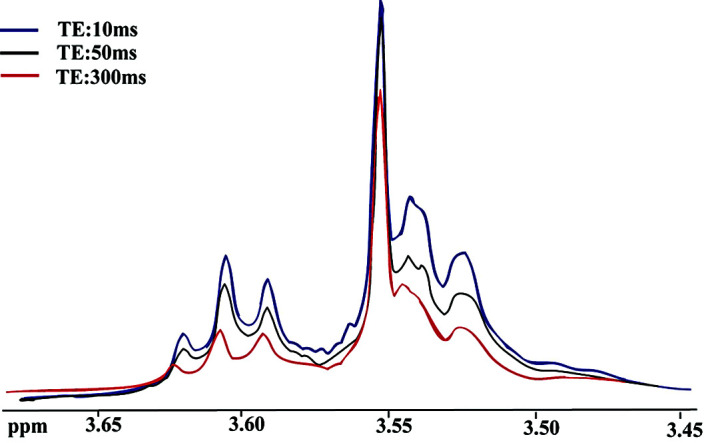
*In vitro* experimental 1D CMPG ^1^H HRMAS spectra of Gly and Myo. The Gly singlet is at 3.55 ppm, the Myo M2 doublet is at 3.53 ppm, and the Myo N2 triplet is at 3.61 ppm.

Fig. 2 shows the results from *in vitro* ^1^H HRMAS experiments on the phantom using 1D CPMG sequence. Our acquisitions ranged from 5 to 480 ms. We thus followed the reduction of Myo signals in favor of the Gly singlet. The Myo signals appeared in phase at a TE of 50 ms, but were reduced with respect to the signals at a TE of 10 ms (not all ^1^H magnetization components were reported in phase for the acquisition). Meanwhile the Gly signal was readily detectable and independent from the Myo signals. When we performed long TE experiments, the Myo signal was further reduced, but the Gly signal was also lost. At a TE of 50 ms, the signal intensity of Myo showed a 50% decrease due to J-modulation.

**Figure 3:**
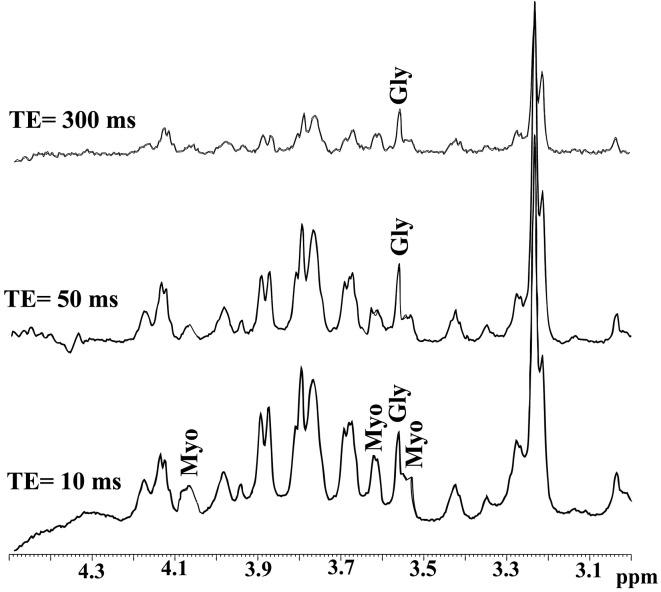
^1^H MR spectra using CPMG from brain metastases obtained at three different TEs. The Gly singlet was detected at 3.55 ppm, and the Myo signals at 3.53, 3.61 and 4.07 ppm. This is a situation where it is important to separate Gly from the overlapping Myo signals since both Gly and Myo are detected at high concentrations (Table I).

**Figure 4:**
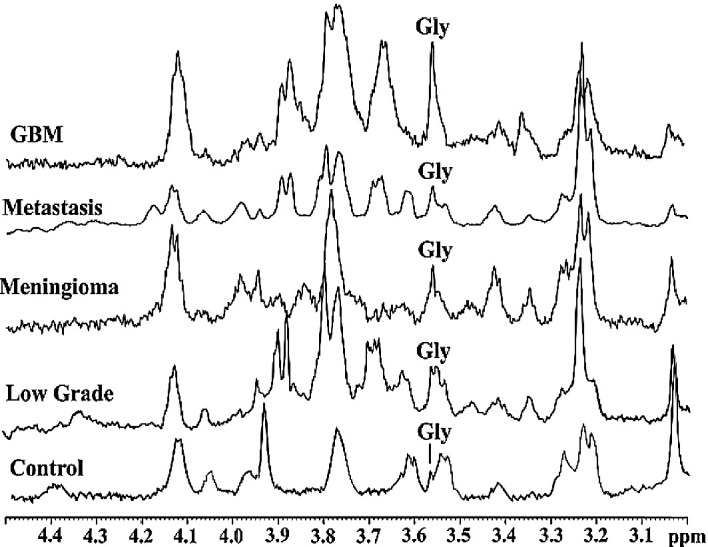
HRMAS ^1^H MR spectra using CPMG in control and brain tumor biopsies acquired with a TE of 50 ms. The Gly singlet at 3.55 ppm is labeled. MR spectra are scaled with respect to TSP signal (not shown here).

As illustrated in Figs. 3 and 4, we demonstrated that detection of Gly in brain tumor biopsies is feasible using HRMAS ^1^H MRS at a TE of 50 ms with CPMG. All spectra were scaled with respect to TSP signal (not shown in figures). The results from the brain biopsies were similar to the phantom data (Fig. 2). Fig. 3 shows MR spectra obtained from brain metastasis samples using 1D CPMG at 3 different TEs. The MT samples showed greater overlap between Myo signals and the Gly singlet at a TE of 10 ms; but when we applied a TE = 50 ms, the Myo signal was greatly reduced and it was possible to obtain a better quantification of Gly metabolites. Using a longer TE (i.e., 300 ms), all metabolite resonances in the spectrum, including the Gly resonance, were reduced.

Fig. 4 shows mean spectra for each tumor type at a TE of 50 ms. The Myo resonance (3.53 ppm) decreased as the TE was increased, and this permitted better Gly detection for quantification purposes. In control brain tissue spectra, the Myo signal dominated the Gly signal. Meanwhile in LG brain tumors, Gly peaks were detectable in spite of the presence of persistent Myo signals. The Gly signal was readily detectable, and almost entirely separate from Myo signals, in the spectra from the other tumor types.

**Figure 5:**
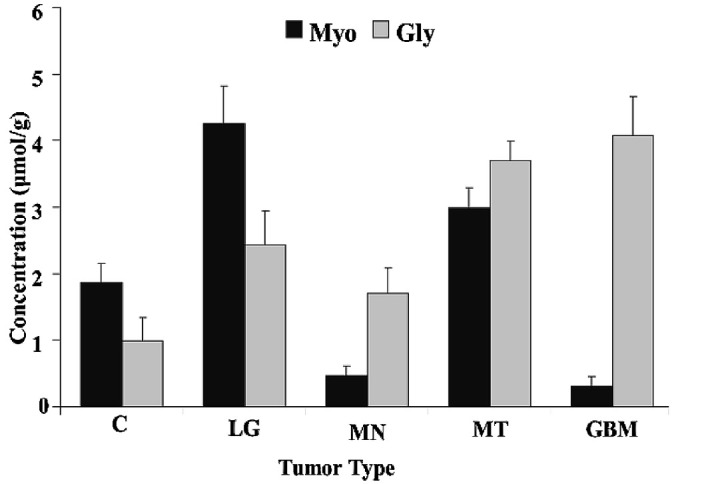
Gly and Myo levels distinguish control from brain tumor biopsies. Note that the major metabolite in GBM tumors was Gly whereas MT tumors contained substantial amounts of Myo. These data provide a clinically important distinction between MT and GBM.

The amounts of Gly and Myo and the Gly:Myo ratios calculated from the CPMG spectra (TE = 50 ms) of control (C) and brain tumor biopsies are summarized in Table I and Fig. 5. Gly levels in all brain tumor types considered as a group were increased relative to control levels. Separate analysis of the different tumor type groups revealed that the mean Gly levels in LG, MT and GBM specimens were significantly higher than control Gly levels (P<0.05). On the other hand, Myo was decreased in GBM and MN tumors relative to control levels (P<0.05).

The mean Gly level in GBM tumors was 67.44% higher than that in LG tumors (P=0.05) and 138.72% higher than that in MN tumors (P=0.01), but did not differ significantly from that in MT tumors (GBM value 10.21% higher, P=0.65 vs. MT). Meanwhile, the mean Myo level in GBM tumors was 92.63% lower than that in LG tumors (P=0.001) and 89.51% lower than that in MT tumors (P=0.0005), but did not differ significantly from that in MN tumors (GBM value 33.17% lower, P=0.465 vs. MN).

The distinction between control and brain tumor biopsies could easily be recognized in observing the relative Gly and Myo levels. The mean Gly:Myo ratio was higher in GBM relative to that in controls (P=0.0004) as well as to the mean ratios of the other tumor types (P≤0.05). The mean Gly:Myo ratio in MN was also higher than the mean control value (P=0.02). It is particularly noteworthy that the mean Gly:Myo ratio for GBM was 889% that for MT (P=0.003), a difference that enabled MT and GBM to be readily distinguishable based on their different Gly:Myo ratios (Fig. 5).

## Discussion

The present study demonstrates that our theoretically and experimentally optimal MRS protocol can be used to detect increased Gly and attenuated Myo in brain tumors. We further demonstrated that the ratio of Gly and Myo detected can be used to discriminate between certain tumor types, namely GBM and MT. A principal finding of our study was that detection of Gly in brain tumor biopsies is feasible using HRMAS ^1^H MRS at a TE of 50 ms with CPMG. We demonstrated the importance of distinguishing Gly from Myo when both metabolites are present at high levels (Fig. 3).

We chose a TE at which the Myo resonance was sufficiently attenuated to allow for optimal Gly detection. If we were to acquire for a longer period of time, we would probably obtain even lower amounts of Myo, but we would also start to lose the Gly signal. We believe that the present tumor classification approach has the advantage of simplicity and is advantageous in terms of not requiring a long time for MR spectral acquisition or sample preparation. Indeed, other approaches (3,17,18) involving data acquisition at long TEs may suffer from substantial signal losses because with increasing magnetic field strength, the Hahn relaxation time T2 of metabolites decreases at high magnetic fields (due to an increased dynamic dephasing contribution). Other methods based on the differential signal dephasing of Gly and Myo that may provide relatively better ability to discriminate healthy brain from brain tumors (19,20) have the probable disadvantage of being time consuming *in vivo*.

Our results showing that we can differentiate brain tumor types based on the amount of Gly and Myo they contain are in agreement with prior observations (31–33). Indeed, the examined tumor types were characterized by differential relative amounts of Gly and Myo (Fig. 5). In agreement with others, we found more Myo in brain tumors than in controls. Indeed, as reported previously (23), we found that Myo was the predominant metabolite over Gly in LG tumors. Such findings may implicate Myo as a marker for tumor malignancy; Myo has also been suggested to be an indicator of reactive astrogliosis (22,23) and of potential astrocytic tumor proliferation rate (34).

Our finding of less Myo in GBM relative to control specimens, however, would be inconsistent with using Myo as a general brain tumor marker. With the data currently available, we cannot definitively explain this discrepancy. It is possible that, in other studies, much of the Gly signal was mis-interpreted as Myo signal due to the overlap between the signals. Our data are in agreement with Castillo *et al*(31) of decreased Myo in GBM relative to controls. However, in the same study, Castillo *et al* found higher Myo levels in LG relative to controls. They explained their findings by suggesting that a lack of activation of the phosphatidylinositol pathway leads to an increased Myo pool in LG tumors that is visible by MRS (31). Thus, we believe that with Gly as a marker for tumor malignancy and Myo as a marker for astrogliosis, distinction between these two metabolites should provide information pertinent to tumor metabolism that is valuable for diagnosis and monitoring of treatment.

We distinguished MN tumors, which had a high Gly:Myo ratio, from LG tumors that had an inverse Gly:Myo ratio (low Gly, high Myo) relative to MN tumors. A low Gly:Myo ratio also distinguishes MT from other tumors, especially GBM, which had the highest ratio of all tumor types examined in this study. The ability to distinguish high-grade gliomas such as GBM from other tumor types (33), especially MT tumors, is clinically important given that our ability to make this distinction is currently inadequate (35). This distinction was not made successfully in a previous study in which GBM and MT were combined into a single group; the authors concluded that a reliable separation between high-grade brain tumors and metastasized tumors cannot be confidently achieved using single voxel ^1^H MRS spectroscopy alone (24–26,35). Here, we demonstrated that reduced Myo together with high Gly can distinguish between GBM and MT, which both exhibit intense, high level Gly signals.

Although our study was performed in brain tissue biopsies *ex vivo*, our approach because of its simplicity, can be easily implemented in high magnetic field clinical scanners *in vivo*, where time constraints are an issue. In the future, *in vivo* MR imaging of gene transcription, when approved for humans, may be an even faster and more specific approach in this regard (36). In fact, in our hands, the gene *cd133*, a stem cell marker for malignant brain tumors (37), seems to be able to discriminate between primary tumors (high or low grade) and metastasized tumors (unpublished data). Thus, *in vivo* MR imaging of the *cd133* gene product may offer a robust clinical approach that could complement the approach proposed herein. Simultaneous MR and positron emission tomography imaging, which is already feasible in humans, is also opening up new possibilities in this area (38).

In conclusion, we demonstrated that by distinguishing Gly from the overlapping Myo signals, we could reliably distinguish between tumor types. The relative presence of Gly and Myo enabled us to distinguish high-grade gliomas from metastasized tumors, a distinction not adequately made at present. We propose that Gly can serve as a useful biomarker in brain tumors.

## Figures and Tables

**Table I:** Concentration of Gly, residual myo-inositol, and Myo:Gly ratio in *μ*mol/g from CPMG spectra (TE = 50 ms) in control and brain tumor biopsies.

Biopsies	Gly	Myo	Gly/Myo
Control			
n=9	0.99±0.34^a^	1.85±0.30	0.54±0.18
LG			
n=10	2.81±0.60	4.25±0.58	0.69±16
% change from C	+183.84	+129.73	+27.78
P-value^b^	0.050	0.04	0.25
MN			
n=7	1.70±0.38	0.46±0.15	3.70±1.07
% change from C	+71.72	−75.14	+585.19
P-value	0.194	0.001	0.02
MT			
n=7	3.07±0.28	2.78±0.31	1.33±1.07
% change from C	+210.10	+50.27	+146.30
P-value	0.0003	0.032	0.03
GBM			
n=12	4.08±0.57	0.31±0.14	13.16±2.24
% change from C	+312.12	−83.24	+2,337.04
P-value	0.001	0.0001	0.0004

a Values are means ±SE;

b Student’s t-test; ND.
